# Ex Vivo Live Full-Thickness Porcine Skin Model as a Versatile In Vitro Testing Method for Skin Barrier Research

**DOI:** 10.3390/ijms22020657

**Published:** 2021-01-11

**Authors:** Jee-hyun Hwang, Haengdueng Jeong, Nahyun Lee, Sumin Hur, Nakyum Lee, Jeong Jun Han, Hye Won Jang, Wang Keun Choi, Ki Taek Nam, Kyung-Min Lim

**Affiliations:** 1 College of Pharmacy, Ewha Womans University, Seodaemungu, Seoul 03760, Korea; cocumuk@naver.com (J.-h.H.); lxxnxhxxx@gmail.com (N.L.); 2 Severance Biomedical Science Institute, Brain Korea 21 PLUS Project for Medical Science, College of Medicine, Yonsei University, Seodaemungu, Seoul 03722, Korea; pwrttony@yuhs.ac (H.J.); blooming93@yuhs.ac (S.H.); nakyum@yahoo.com (N.L.); 3 GF Fermentech Inc., Sejong-Si 30077, Korea; jjhan@genofocus.com; 4 LCS Biotech, Suwon-Si 16614, Korea; raneehw@lcsbio.com (H.W.J.); wkchoi@lcsbio.com (W.K.C.)

**Keywords:** ex vivo skin model, hydroxyacids, skin barrier, skin permeability, stratum corneum

## Abstract

Since the European Union (EU) announced their animal testing ban in 2013, all animal experiments related to cosmetics have been prohibited, creating a demand for alternatives to animal experiments for skin studies. Here, we investigated whether an ex vivo live porcine skin model can be employed to study the safety and skin barrier-improving effects of hydroxyacids widely used in cosmetics for keratolytic peels. Glycolic acid (1–10%), salicylic acid (0.2–2%), and lactobionic acid (1.2–12%) were used as representative substances for α-hydroxyacid (AHA), β-hydroxyacid (BHA), and polyhydroxyacid (PHA), respectively. When hydroxyacids were applied at high concentrations on the porcine skin every other day for 6 days, tissue viability was reduced to 50–80%, suggesting that the toxicity of cosmetic ingredients can be evaluated with this model. Based on tissue viability, the treatment scheme was changed to a single exposure for 20 min. The protective effects of a single exposure of hydroxyacids on skin barrier function were evaluated by examining rhodamine permeability and epidermal structural components of barrier function using immunohistochemistry (IHC) and immunofluorescence (IF) staining. Lactobionic acid (PHAs) improved skin barrier function most compared to other AHAs and BHAs. Most importantly, trans-epidermal water loss (TEWL), an important functional marker of skin barrier function, could be measured with this model, which confirmed the significant skin barrier-protective effects of PHAs. Collectively, we demonstrated that the ex vivo live full-thickness porcine skin model can be an excellent alternative to animal experiments for skin studies on the safety and efficacy of cosmetic ingredients.

## 1. Introduction

The skin is an organ composed of epidermis, dermis, and subcutaneous tissue. The skin is only millimeters thick, but works as an efficient barrier to protect the body from moisture loss as well as the permeation of exogenous substances such as pathogens [1] and chemicals. As increasing attention is given to beauty in modern society, the skin has become one of the leading research topics. Previously, most dermatological research was performed on experimental animals such as mice [2], rats [3], and rabbits. However, since the EU announced their animal testing ban in 2013, animal experiments on cosmetics have been prohibited, demanding the development of alternatives to animal experiments for skin research.

To date, several alternative testing methods have been developed for skin studies. Of these, living skin equivalents, such as reconstructed human epidermis (RHE), reconstructed full-thickness (FT) human skin, and skin organ culture (SOC) models have been investigated as alternatives to animal experiments to test the efficacy and safety of new cosmetic ingredients or finished cosmetic products. These models not only allow compliance with animal testing bans but also provide the means to improve and extend our knowledge of skin biology. Moreover, they have proven to be efficient, informative, and predictive tools for screening, bioavailability, and efficacy testing of active ingredients, and can serve as a preclinical safety model, which is very informative in the design of clinical testing [4].

One of the main advantages of reconstructed human skin (RHS) models is that they can mimic the function of real human skin in vivo, as they have a fully differentiated epidermis. The presence of the stratum corneum (SC) on the air-liquid interface enables topical application of both aqueous and oil solutions as well as final cosmetic formulations. However, RHS models have some inherent limitations, such as impaired barrier function due to incomplete maturation of the stratum corneum, and often require extended cultivation procedures with costly media. Most importantly, the FT model is complicated to make and accordingly costly. In this context, human SOC models are getting enormous attention from cosmetic researchers. Human skin explants are easy to prepare, simple to handle, exhibit good barrier function, and inherently have complete dermal and epidermal structure. However, human SOC is difficult to obtain for mass production as it involves informed consent from the skin donors and shows substantial inter-individual variability, which significantly limits wider application [4].

The pertinence of porcine to human skin from both histological and physiological viewpoints is been well-established [5]. Porcine skin can be obtained relatively cheaply as a by-product from slaughterhouses without sacrificing extra animals and, in this particular context [6], does not contradict the animal testing ban, like bovine corneal opacity tests or chick eye tests. It is also possible to control the age of the animals as well as the body site from which the skin biopsy was taken, which is critical for ensuring reproducibility [5]. In this regard, the ex vivo porcine skin model may be an ideal replacement for human skin models.

**Figure 1:**
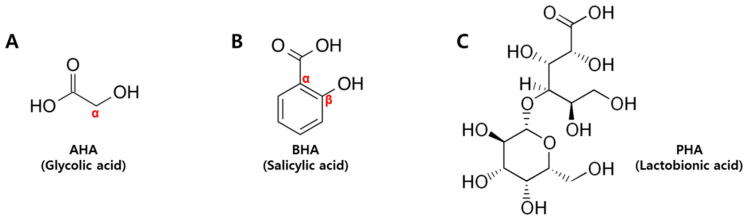
Classification of hydroxyacids used in this study (**A**) AHA (glycolic acid), (**B**) BHA (salicylic acid), and (**C**) PHA (lactobionic acid). AHA: α-hydroxyacid, BHA: β-hydroxyacid, PHA: polyhydroxyacid.

Here, we investigated whether the ex vivo porcine skin model can be used for studies on skin barrier function. We used the hydroxyacids widely employed in cosmetic and drug formulations to control excessive cornification and to test whether the ex vivo porcine skin model can be used to evaluate the safety and effects of cosmetic ingredients. α-Hydroxyacids (AHAs) are organic carboxylic acids with one hydroxyl group attached directly to the α position of an aliphatic or alicyclic carbon atom, but not to a benzene or other aromatic ring. On a broader scope, AHAs may include those molecules that have additional carboxyl groups [7]. Glycolic acid, present in sugar cane juice, is the smallest molecule of all the hydroxyacids, and is a major ingredient in most AHA products on the market. All other AHAs may be considered derivatives or substituted glycolic acid [8] (Figure 1A). β-Hydroxyacids (BHAs) are organic carboxylic acids with one hydroxyl group attached to a carbon atom at the β-position, and are represented by β-hydroxybutanoic acid and tropic acid. Salicylic acid, or 2-hydroxybenzoic acid, has both hydroxyl and carboxyl groups directly attached to a benzene ring. It is not chemically a true BHA, but it is erroneously referred to as a BHA in casual jargon [9] (Figure 1B). Polyhydroxyacids (PHAs) are organic carboxylic acids with multiple hydroxyl groups. Many PHAs are also AHAs; they are derived from carbohydrates and are important intermediates in carbohydrate metabolism [8]. In particular, aldobionic acid (ABA) consists of one monosaccharide chemically linked through an ether bond to an aldonic acid. An aldobionic acid may also be described as an oxidized form of a disaccharide or dimeric carbohydrate, such as lactobionic acid, which is derived from lactose [8] (Figure 1C).

In order to examine whether the ex vivo porcine skin model can be used for the evaluation of the safety of hydroxyacids, these acids were applied to the ex vivo porcine skin and tissue viability was assessed with MTT assay and histology. Epidermal structural components of barrier function were investigated using immunohistochemistry and immunofluorescence staining. The effects of hydroxyacids on barrier function were evaluated by measuring rhodamine permeability and trans-epidermal water loss (TEWL).

## 2. Results

### 2.1. Assessment of Damage to Ex Vivo Porcine Skin Inflicted by Repeated Application of High-Concentration Hydroxyacids

**Figure 2:**
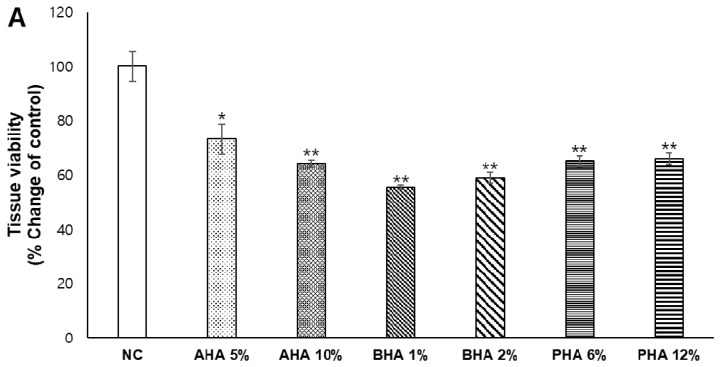

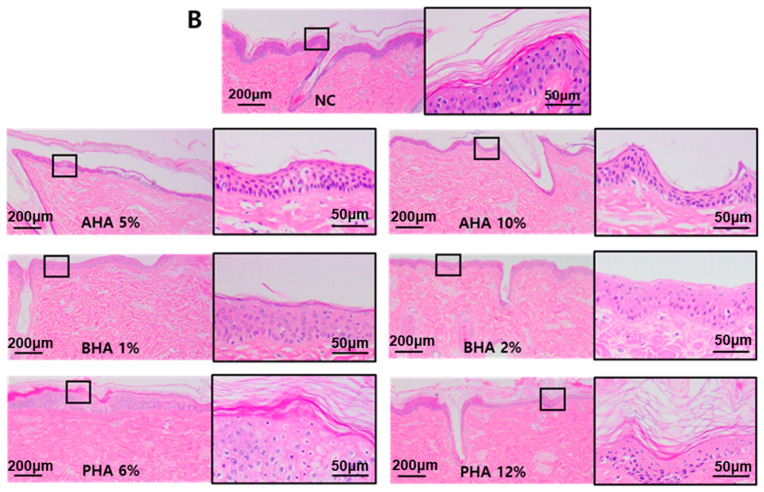
Damage to ex vivo porcine skin after repeated application of hydroxyacids at high concentrations Hydroxyacids were applied at high concentrations on the porcine skin every other day for six days: AHA, 5–10%; BHA, 1–2%; and PHA, 6–12%. (**A**) Tissue viability of the treated ex vivo porcine skin was determined using the WST-1 assay. Values are mean ± SE (*n* ≥ 3). Significant differences are denoted by * *p* < 0.05 and ** *p* < 0.01. (**B**) Porcine skin was processed and H&E stained. A representative microphotograph is shown. NC; negative control (30% ethanol).

Hydroxyacids are inherently irritating to the skin when applied at high concentrations or after repeated exposure [10]. In order to examine whether the ex vivo porcine skin model could be used to evaluate the safety of cosmetic ingredients, hydroxyacids were applied at high concentrations on the porcine skin every other day for 6 days as follows: AHA, 5–10% (0.66–1.32 mM); BHA, 1–2% (0.08–0.15 mM); and PHA, 6–12% (0.17–0.34 mM), based on the generally used concentrations in chemical peels. Then, tissue viability was determined using the WST-1 assay. All the hydroxyacids reduced tissue viability to 50–80% compared to the control group (Figure 2A). In addition, when the treated porcine skin tissues were processed and stained with hematoxylin and eosin (H&E), it was evident that the stratum corneum (SC) was severely damaged by the application of AHA and BHA along with vacuolation and derangement of keratinocytes, and spongiosis of the epidermis. In the case of PHA, the degree of damage was less severe than that of other hydroxyacids (Figure 2B).

### 2.2. Effects of a Single Application of Hydroxyacids at Low Concentrations on the Tissue Integrity of Ex Vivo Porcine Skin

**Figure 3:**
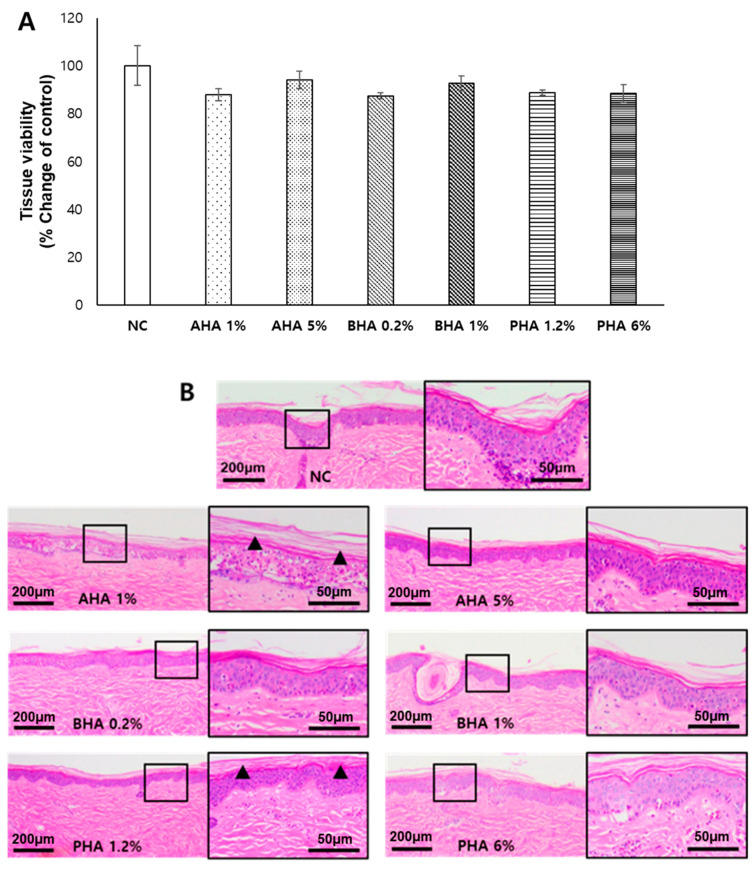
Effects of a single treatment with low-concentration hydroxyacids on ex vivo porcine skin Hydroxyacids were applied once for 20 min at low concentrations: AHA, 1–5%; BHA, 0.2–1%; and PHA, 1.2–6%. The ex vivo porcine skin was washed and incubated for six days. (**A**) Tissue viability of the treated ex vivo porcine skin was determined using the WST-1 assay. Values are mean ± SE (*n* ≥ 3). (**B**) Porcine skin was processed and H&E stained. A representative microphotograph is shown. NC; negative control (30% ethanol). The area in which the stratum corneum is well-organized compared to the negative control is indicated by black arrow heads.

Generally, chemical peels are used as a single treatment procedure to prevent skin damage. Based on the tissue viability data above, the treatment scheme was changed to a single exposure for 20 min. In addition, the concentration of hydroxyacids was lowered to 1–5% (0.13–0.66 mM) for AHA, 0.2–1% (0.02–0.08 mM) for BHA, and 1.2–6% (0.03–0.17 mM) for PHA. After six days of incubation with media changes after the treatment, tissue viability was not significantly reduced by hydroxyacids compared to the negative control, suggesting that hydroxyacids at these concentrations may not cause skin irritation (Figure 3A). On histological analysis, the stratified structures of the stratum corneum (SC), stratum granulosum, stratum spinosum, and stratum basale were relatively well preserved. Interestingly, there was a marked improvement in the structural integrity of the SC after treatment with hydroxyacids. Generally, the SC exhibited more well-organized features, showing even and flat stacking after hydroxyacids were applied. These effects were more remarkable at lower concentrations of AHA (glycolic acid 1%) and PHA (lactobionic acid 1.2%) (Figure 3B).

### 2.3. Effect of Hydroxyacids on the Skin Penetration of Rhodamine B

**Figure 4:**
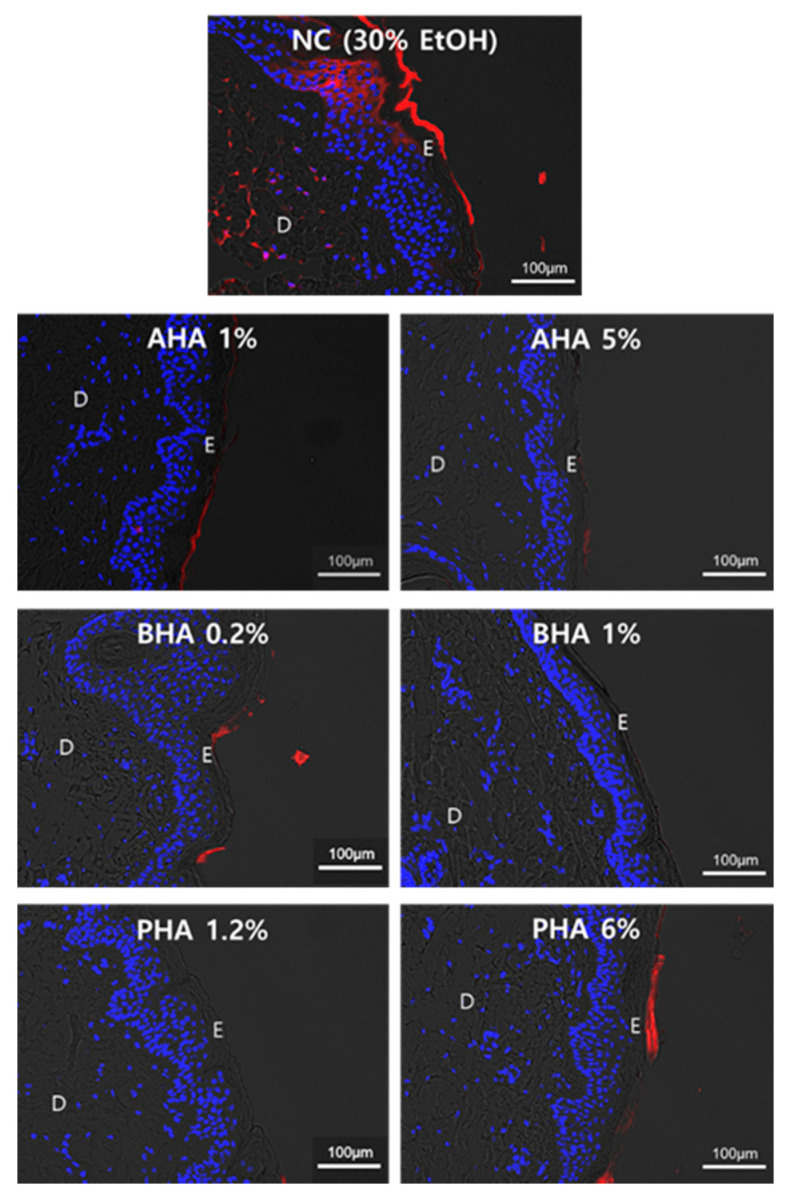
Effect of hydroxyacids on the skin penetration of rhodamine B Permeability of rhodamine B was evaluated after 24-h incubation of treated tissue. The fluorescent images represent rhodamine B (red) and DAPI (blue) staining. D: Dermis, E: Epidermis.

We evaluated the skin barrier protection effect of hydroxyacids by determining the skin penetration of rhodamine B (Rb). Rb, a fluorescent dye, was used to evaluate the permeability of the skin. Rb was selected as it is a poorly absorbed dye and one of the most widely used fluorescent probes in biomedical applications, including skin permeation studies [11,12]. As shown in the fluorescence image, the epidermis and the dermis were clearly distinguished. In the negative control, Rb (red) penetrated deeply into the dermis through the epidermis. However, after treatment with hydroxyacids, Rb did not reach all the dermis, and its penetration was retained within the SC. This is likely due to the well-ordered SC after treatment with hydroxyacids, which confirmed the results of H&E staining (Figure 4).

### 2.4. Effect of Hydroxyacids on the Skin Barrier Function as Measured by TEWL

**Figure 5:**
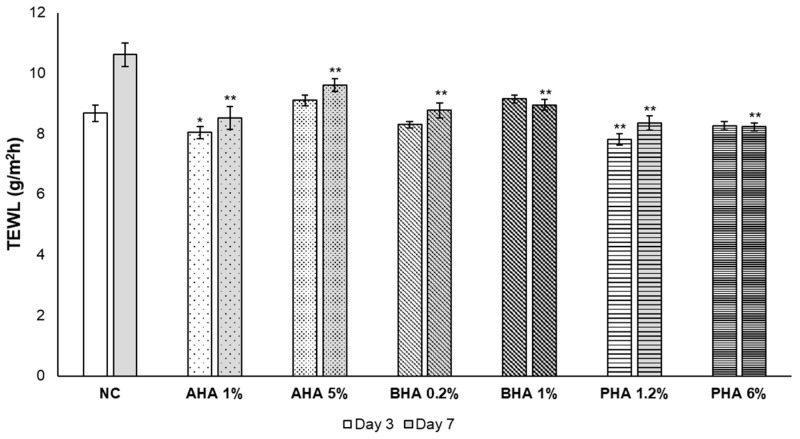
Effect of hydroxyacids on skin barrier function. Measurement using a vapometer with a closed chamber. Measurements were consecutively repeated three times for each skin biopsy. The mean of the three measurements was used as a representative value. Significant differences are denoted by * *p* < 0.05 and ** *p* < 0.01.

Free water from viable tissue continually diffuses through the SC, evaporating from its surface. This is known as trans-epidermal water loss (TEWL). TEWL measurement is well established, noninvasive, and widely used to assess functional changes in skin barrier properties [13]. Measurement of TEWL was performed on days 3 and 7 after the single hydroxyacid treatment using a vapometer with a closed chamber. In most cases, TEWL increased over time. However, on the 3rd day, TEWL was significantly lower in the tissues treated with low concentrations of AHA (glycolic acid 1%) and PHA (lactobionic acid 1.2%) compared to the negative control, which supported the histopathological results and Rb penetration study. In addition, on the 7th day, it was confirmed that TEWL was significantly lower than that of the control group for all the treated hydroxyacids, suggesting that a single treatment procedure with hydroxyacids may have enhanced the barrier function of the SC (Figure 5).

### 2.5. Immunohistochemical and Immunofluorescence Staining of Key Protein Components of the Skin Epidermis

**Figure 6:**
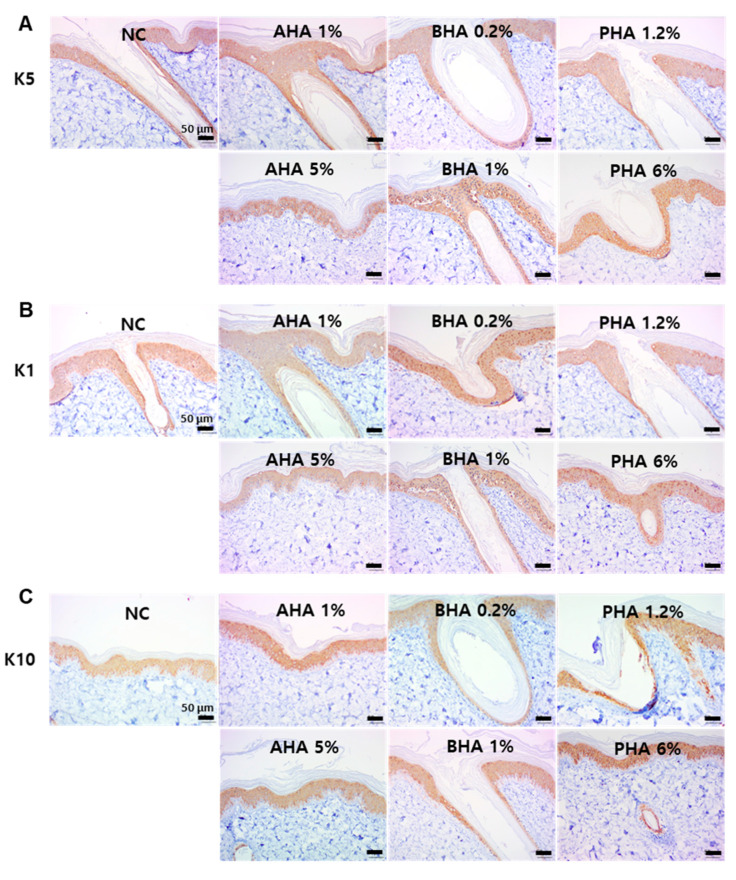

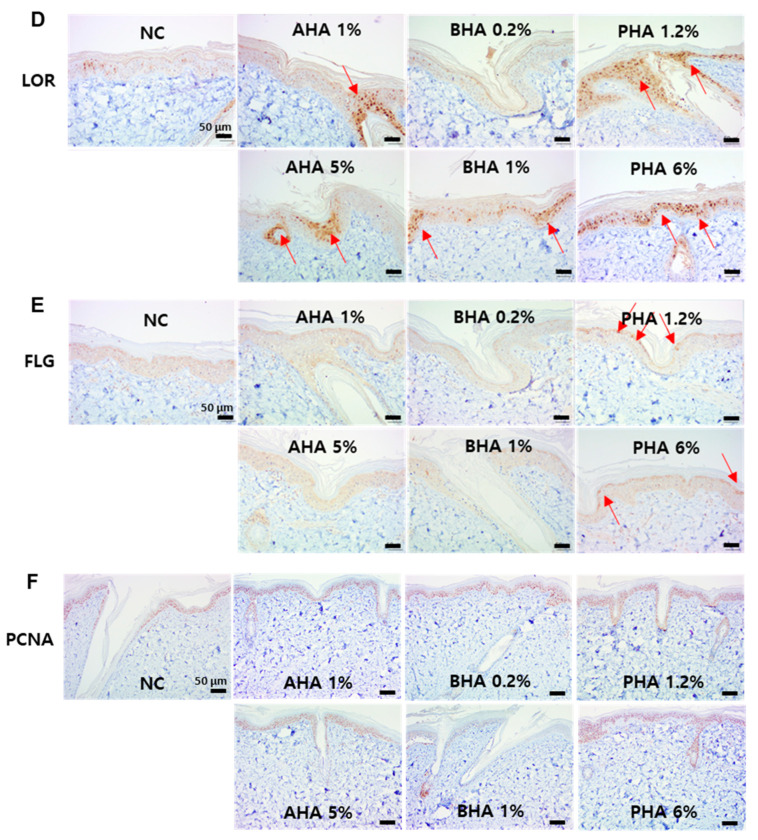
Immunohistochemical staining of key protein components of the skin epidermis Immunohistochemical images of ex vivo porcine skin with antibodies against (**A**) K5, (**B**) K1, (**C**) K10, (**D**) LOR, (**E**) FLG, and (**F**) PCNA. K1: Keratin 1, K5: Keratin 5, K10: Keratin 10, LOR: loricrin, FLG: filaggrin, PCNA: proliferating cell nuclear antigen. The regions in which the expression of the specific protein is significantly higher than the negative control are indicated by red arrows. All scale bar represents 50 μm.

Keratin forms tonofilaments that act as a cytoskeleton to maintain the structure of the keratinocyte. When keratinized, keratin fibers in the granular cell layer aggregate with the help of filaggrin to form the characteristic condensed keratin pattern. Loricrin is a major protein component of the cornified cell envelope found in terminally differentiated epidermal cells [14]. After the single treatment with several hydroxyacids, immunohistochemical (IHC) images were obtained using antibodies against keratin (K) 5 (basal layer), 1, 10 (suprabasal layer), loricrin, filaggrin, and PCNA (Figure 6). We found that although nonspecific staining prevented clear interpretation, the treatment with hydroxyacids did not significantly affect keratins (K5, K1, and K10) (Figure 6A–C). However, the expression of loricrin was remarkably increased by hydroxyacids (Figure 6D). In addition, the expression of filaggrin was increased with the application of some hydroxyacids, especially PHA (lactobionic acid 6% and 1.2%) (Figure 6E). Cell proliferation as indicated by PCNA staining also increased after the treatment with hydroxyacids, except for BHA 1% (Figure 6F).

**Figure 7:**
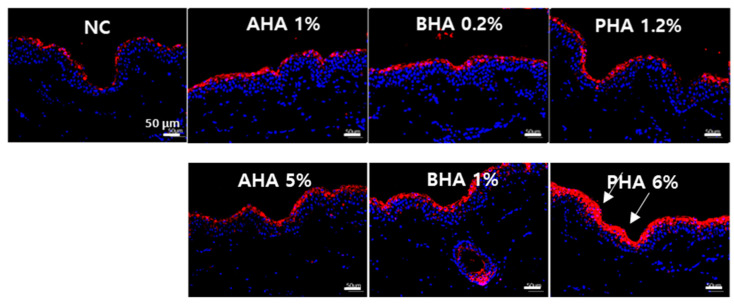
Immunofluorescence staining of tight junction protein, claudin 1, in the skin epidermis. Immunofluorescence images of ex vivo porcine skin with antibodies against tight junction protein, CLDN1: claudin 1 is shown in red and DAPI is shown in blue.

Claudins and occludin are the most important components of the tight junctions (zonulae occludentes). Tight junctions establish the paracellular barrier that controls the flow of molecules in the intercellular space between epithelial cells [15]. In order to investigate the effect of hydroxyacids on tight junctions, we obtained IF images for claudin 1 and occludin. While occludin staining was negative in the porcine skin, claudin 1 (red) was increased after treatment with a high concentration of PHA (lactobionic acid 6%) (Figure 7). 

**Figure 8:**
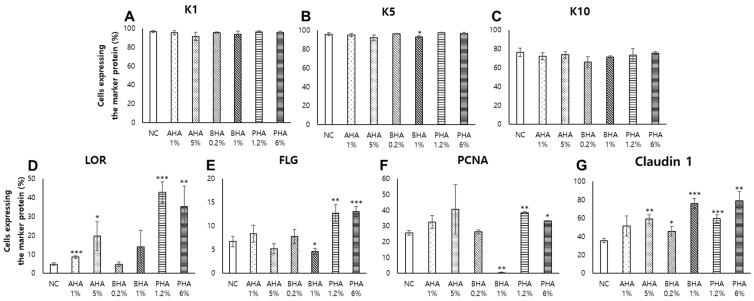
Quantification of the cells expressing marker proteins in immunohistochemistry and immunofluorescence images. 3,3′-Diaminobenzidine (DAB)-positive pixels were quantified in the epidermis excluding the hair follicle region using Qupath software [16]. The DAB positive pixels were normalized with total stained pixel counts (hematoxylin + DAB) in IHC or DAPI positive pixels in IF to calculate the percentage of cells expressing the marker protein. (**A**) K1, (**B**) K5, (**C**) K10, (**D**) LOR, (**E**) FLG, (**F**) PCNA, and (**G**) Claudin 1. Data are presented as the mean ± SEM (*n* = 3–4 fields per a group). *p* < 0.05 was considered significant (Student’s *t* test vs NC; * *p* < 0.05; ** *p* < 0.01; *** *p* < 0.001).

Quantification of the percentages of cell population expressing the marker proteins in the skin epidermis excluding the hair follicle region of IHC and IF images, is shown in Figure 8. It can be seen that the application of PHA (with AHA or BHA less pronounced) significantly increased the cell populations expressing loricrin (~8.8 fold) (Figure 8D), filaggrin (~2.0 fold) (Figure 8E), PCNA (~1.5 fold) (Figure 8F) and claudin (~2.2 fold) (Figure 8G) while keratins were relatively unaffected (Figure 8A–C), suggesting that PHA may augment the epidermal cell regeneration and skin barrier protein expression.

## 3. Discussion

The epidermis protects organisms against mechanical injury and dehydration and regulates immune homeostasis by virtue of epidermal keratinocytes. The epidermal barrier is formed by keratinocytes contributing tight junctions and the cornified envelope and by Langerhans cells providing immune functions [17]. Barrier dysfunction and cutaneous sensitization can give rise to chronic inflammatory disorders, including atopic dermatitis and psoriasis [18]. In this context, the skin barrier has been a focus of intense studies with the goal of discovering novel agents that can improve this barrier. Therefore, versatile skin models with a wide range of applications that can be used for the study of various chemicals and endpoints are pivotal. We employed keratolytic hydroxyacids to investigate whether the ex vivo porcine skin model can be used to evaluate the safety and efficacy of cosmetic ingredients in improving skin barrier function. Since these keratolytic hydroxyacids have low pH, cell line models or even reconstructed skin models are not appropriate due to nonspecific toxicity caused by acidity. The ex vivo porcine skin model, which has a robust stratum corneum, is therefore useful in the assessment of the safety and efficacy of the hydroxyacids. In addition to acids, there are many chemicals that cannot be tested in conventional in vitro models, such as organic solvents, substances with non-soluble physical states, or substances with a certain level of nonspecific toxicity. Actually, many cosmetic ingredients and formulations belong to these substances, and are often untestable in vitro. In these cases, an ex vivo porcine skin model would be an excellent option that warrants further studies in the future.

As the porcine skin model has similar properties to human skin, it has recently been used in several studies on ultraviolet ray-induced skin damages [19], wound healing [20,21,22], and drug delivery [23,24]. In these studies, endpoints such as tissue viability and histopathology were used to evaluate skin damage. In this study, in addition to viability and histopathology, we showed that various endpoints such as skin permeability using a fluorescent dye, TEWL, IHC, and IF could be evaluated, which are important for various skin studies. Porcine skin is readily obtainable from abattoirs without sacrificing extra animals for research. Moreover, the similarity of porcine skin to human skin is well established [25,26,27,28,29,30]. Studies examining the thickness of various skin layers have shown that the SC thickness is 21–26 μm in pigs, which is comparable to that of human skin [27,31,32]. The follicular structure of pig skin also resembles that of humans, with hairs and infundibula extending deeply into the dermis [27]. Moreover, the vascular anatomy and collagen fiber arrangement in the dermis, as well as the contents of SC glycosphingolipids and ceramides, are similar to those of the human [33], supporting the utility of the ex vivo porcine skin model for studying the physiology of human skin.

In this study, glycolic acid (1–10%), salicylic acid (0.2–2%), and lactobionic acid (1.2–12%) were used as the representative substances for AHA, BHA, and PHA, respectively. When hydroxyacids were applied at high concentrations on the porcine skin every other day for six days, tissue viability was reduced to 50–80%, suggesting that the toxicity of cosmetic ingredients could be evaluated with this model. Based on tissue viability, the treatment scheme was changed to a single exposure for 20 min. As a result, there was no significant difference in tissue viability compared to the negative control; that is, the tested concentration of hydroxyacids did not cause skin irritation. Histological analysis confirmed that the SC of the epidermis became well ordered without any damages when treated with lower concentrations of AHA and PHA. The permeation of the fluorescent dye Rb was also decreased significantly when hydroxyacids were applied. TEWL measured on the 3rd and 7th days after treatment with hydroxyacids also confirmed the skin barrier-protective effects of PHAs.

Of note, when PHA was applied, the expression of loricrin was greatly increased. PHA also increased the expression of filaggrin, claudin and PCNA as can be clearly seen in IHC, and IF images and quantification of marker protein expressing cells, supporting that improved skin barrier function following PHA treatment may stem from the enhancement of epidermal cell regeneration and skin barrier protein expression. Even when compared to other hydroxyacids, PHA, showed the most remarkable effects on skin barrier function. A distinctive feature of PHA compared to AHA and BHA is suggested to be the presence of numerous hydroxyl groups and chelating properties. Due to this, PHAs are capable of preserving the water content in the epidermis and therefore have an excellent moisturizing effect [34]. In addition, PHAs have strong antioxidant properties owing to their ability to chelate metal ions, and therefore can prevent the oxidation of unstable substances such as anthralin and hydroquinone [35], suggesting that PHAs might be an ideal cosmetic ingredient for a safe chemical peel.

Hydroxyacids have been used, typically in concentrations ranging from 2% to 70%, to treat acne, ichthyosis, keratoses, warts, psoriasis, photoaged skin, and other skin disorders [36]. Hydroxyacids induce desquamation of epidermis by enhancing breakdown and decreasing cohesiveness of corneosomes. Hydroxyacids also increase epidermal cell renewal and epidermal enzyme activities, leading to epidermolysis and exfoliation [37]. These effects of hydroxyacids ultimately result in the improvement of skin texture and coloration and clearing of pores [38]. In line with these reports, our study revealed that hydroxyacids improved the stratum corneum arrangement, increased basal cell proliferation and skin barrier protein expression. Furthermore, safety issues of hydroxyacids such as erythema [39,40] can be captured well by our model as determined by reduced tissue viability, stratum corneum dilapidation and epidermal cell death upon repeated application of hydroxyacids at high concentrations. This suggests that the ex vivo full-thickness live porcine skin model can be used to evaluate the efficacy and safety of cosmetic ingredients.

Collectively, we demonstrated that the ex vivo live full-thickness porcine skin model could be an excellent alternative to animal experiments for skin studies on the safety and efficacy of cosmetic ingredients. We showed that ex vivo live full-thickness porcine skin model can be used for skin barrier studies by providing various endpoints such as viability, histology, and functional assessment. It would be interesting whether the ex vivo porcine skin model can be extended to evaluate other cosmetic efficacy and safety studies that include whitening, anti-aging, and hair health, which warrants future research.

## 4. Materials and Methods

### 4.1. Materials and Reagents

Glycolic acid (70%) (representing α-hydroxyacids [AHA]) was purchased from Ajinomoto Co., Inc. (Tokyo, Japan), salicylic acid (99%) (representing β-hydroxyacids [BHA]) was purchased from Junsei Chemical Co., Ltd. (Tokyo, Japan), and lactobionic acid (representing polyhydroxyacid [PHA]) was purchased from GF Fermentech, Inc. (Sejong-si, Korea). These were diluted with 30% ethanol. Phosphate-buffered saline (PBS), sodium dodecyl sulfate (SDS), and rhodamine B were purchased from Sigma-Aldrich (St. Louis, MO, USA). WST-1 (4-[3-(indophenyl)-2-(4-nitrophenyl)-2H-5-tetrazolio]-1,3-benzene disulfonate) was purchased from Roche (Indianapolis, IN, USA).

### 4.2. Ex Vivo Live Full-Thickness Porcine Skin

The porcine skins were obtained from Apures Co. (Gyeonggi-do, Korea). All porcine skins were obtained from the back of Micropig, which was sacrificed for research on drug delivery. Skin tissue (2 × 2 cm^2^) from which the lipid layer had been removed was rinsed with 70% ethanol and PBS. Nine punch biopsies were taken from each tissue. Biopsy diameters of 6 mm were used. After placing the 3D-printed plastic mesh on a 24-well plate, the biopsy skin was placed on the mesh. After properly filling the culture media, the tissues were pre-incubated for 20–24 h at 37 °C in a humidified atmosphere containing 5% CO_2_. After pre-incubation, the tissues were treated with test materials diluted in 30% ethanol. After 20 min of treatment, tissues were rinsed with PBS and cultured. Culture media was changed every 48 h.

### 4.3. WST-1 Assay

Tissue viability was determined by the WST-1 assay, which does less damage to skin tissues than MTT. After incubation, 300 μL of WST-1 diluted in sterile PBS were transferred into a 96-well plate, and absorbance was determined by microplate spectrophotometer readings at 450 nm (BioTek Instruments, Inc., Winooski, VT, USA). Tissue viability of the skin sample was expressed as the ratio of the skin disk to its weight in milligrams.

### 4.4. Histological Analysis

For the histological examination, biopsy skin samples were fixed in 10% neutral-buffered formalin. Preserved tissues from each group were paraffin wax-embedded, sectioned, stained with hematoxylin and eosin (H&E), and then examined microscopically under an Olympus DP71 microscope (Center Valley, PA, USA). All tissue images were obtained using the virtual slide system (Aperio Scanscope XT, Vista, CA, USA).

### 4.5. Skin Penetration Study

Rhodamine B, a hydrophilic dye, was applied to evaluate the permeability of the skin as described previously [41]. Treated tissues were incubated for 24 h and rinsed with PBS. 0.02% rhodamine B was applied for 2 h and gently washed with an autoclaved cotton swab. Then, cultured tissues were initially cut in 100-mm sections and immediately embedded in OCT compound (Sakura, Tokyo, Japan) using dry ice. Embedded samples were cut into 5-μm sections and incubated at room temperature for 40–60 min. Samples were washed in PBS twice for 3 min. After washing, 1:1,000 DAPI (Sigma, St. Louis, OU, USA) in PBS was applied for 5 min on slides which were pre-marked by a pen (Enzo, ADI-950-233-0001, Farmingdale, IL, USA), followed by three washes in PBS. Then, samples were covered by mounting solution (Thermo Scientific, Waltham, MA, USA), and immunofluorescence images were captured by EVOS-FL (Thermo Scientific, Fremont, CA, USA).

### 4.6. Immunohistochemistry (IHC) and Immunofluorescence (IF) Staining

For immunostaining, paraffin-embedded pig skins were cut into 4-μm sections. The sections were de-paraffinized in xylene and sequentially rehydrated through a descending graded series (100%, 95%, and 70%) of ethanol. Antigen retrieval (DAKO, S1699, Santa Clara, CA, USA) was conducted using a high-pressure cooker for 15 min. After cooling on ice until the solution became transparent, sections were incubated in 3% H_2_O_2_ for 30 min and washed twice with PBS. To reduce nonspecific signals, samples were incubated with serum-free protein blocking solution (DAKO, X0909, Santa Clara, CA, USA) for 1–2 h at room temperature. Anti-keratin1 (KRT1) (abcam, ab185628, 1:1000, Cambridge, UK), anti-KRT5 (abcam, ab52635, 1:500, Cambridge, UK), anti-KRT10 (abcam, ab76318, 1:1000, Cambridge, UK), anti-loricrin (LOR) (abcam, ab85679, 1:1000, Cambridge, UK), anti-filaggrin (FLG) (Biolegend, #905804, 1:1000, San Diego, CA, USA), or anti-PCNA (clone PC10; Santa Cruz Biotechnology, Dallas, TX, USA) were incubated overnight at 4 °C in a humidity chamber. After three washes in PBS, sections were incubated in HRP-conjugated anti-rabbit secondary antibody (DAKO, K4003, Santa Clara, CA, USA) for 15 min at room temperature. For immunohistochemistry, DAB (DAKO, K3468, Santa Clara, CA, USA) was used for the development of antibodies, and Mayer’s hematoxylin (DAKO, S3309, Santa Clara, CA, USA) was used for counterstaining. Each experiment was performed using an identical time for DAB development.

For immunofluorescence, anti-Claudin1 (CST, #13255, 1:1000, Danvers, MA, USA) was detected with cy3-conjugated anti-rabbit IgG (Invitrogen, Carlsbad, CA, USA), and DAPI was used for nuclear staining. Immunofluorescence images were taken with an EVOS-FL.

### 4.7. Quantitation of Immunohistochemistry (IHC) and Immunofluorescence (IF) Staining

Qupath software [16] was utilized to quantify immunohistochemistry (IHC) and immunofluorescence (IF) images. To measure DAB-positive pixels representing the cells expressing the marker protein in IHC, we ran the ‘positive pixel count’ module built in the software and calculated the positive pixels above the threshold in the skin epidermis in 20X high power fields. The DAB positive pixel percentage was normalized with total stained pixel (hematoxylin + DAB) counts. To measure the claudin positive cells in the IF images, we ran the ‘positive pixel detection’ module and calculated the positive cells above the threshold in skin epidermis in 20X high power field. Positive cell percentage was normalized with total DAPI positive cells such that the values representing the portion of cells expressing the specified marker protein. Thresholds for DAB or cy3-staining of each marker were K1 = 0.1, K5 = 0.1, K10 = 0.2, LOR = 0.2, FLG = 0.2, PCNA = 0.28, and Claudin = 50, respectively.

### 4.8. Trans-Epidermal Water Loss (TEWL)

TEWL was measured using a VapoMeter SWL4001TJ (Delfin Technologies Ltd., Kuopio, Finland), a portable, battery-operated, closed, unventilated chamber evaporimeter. Measurements were consecutively repeated three times for each skin biopsy. The mean of the three measurements was used as a representative value.

### 4.9. Statistical Analysis

Results are expressed as the mean ± standard error of the mean (SEM) of three or more independent experiments. The statistical analyses were performed with the Student’s t-test or two-way ANOVA. *p*-values < 0.05 were considered statistically significant.

## Data Availability

The data presented in this study are available on request from the corresponding author. The data are not publicly available since they are raw data.

## Abbreviations

AHA	α-hydroxyacid
BHA	β-hydroxyacid
PHA	Polyhydroxyacid
TEWL	Trans-epidermal water loss
Rb	Rhodamine B
D	Dermis
E	Epidermis
IHC	Immunohistochemistry
IF	Immunofluorescence
K1	Keratin 1
K5	Keratin 5
K10	Keratin 10
LOR	Loricrin
FLG	Filaggrin
CLDN1	Claudin 1
WST-1	(4-[3-(indophenyl)-2-(4-nitrophenyl)-2H-5-tetrazolio]-1,3-benzene disulfonate)
NC	Negative control
SC	Stratum corneum
SOC	Skin organ culture
PCNA	Proliferating cell nuclear antigen.
